# Supplementary material for: Rhizosheath inhabiting Massilia are linked to heterosis in roots of maize
Source: Nat Commun. 2025 Nov 28;16:10777. doi: 10.1038/s41467-025-65829-2 (PMC12663149; doi:10.1038/s41467-025-65829-2)
Supplement: Supplementary file 1 — Supplementary Information [file 41467_2025_65829_MOESM1_ESM.pdf]

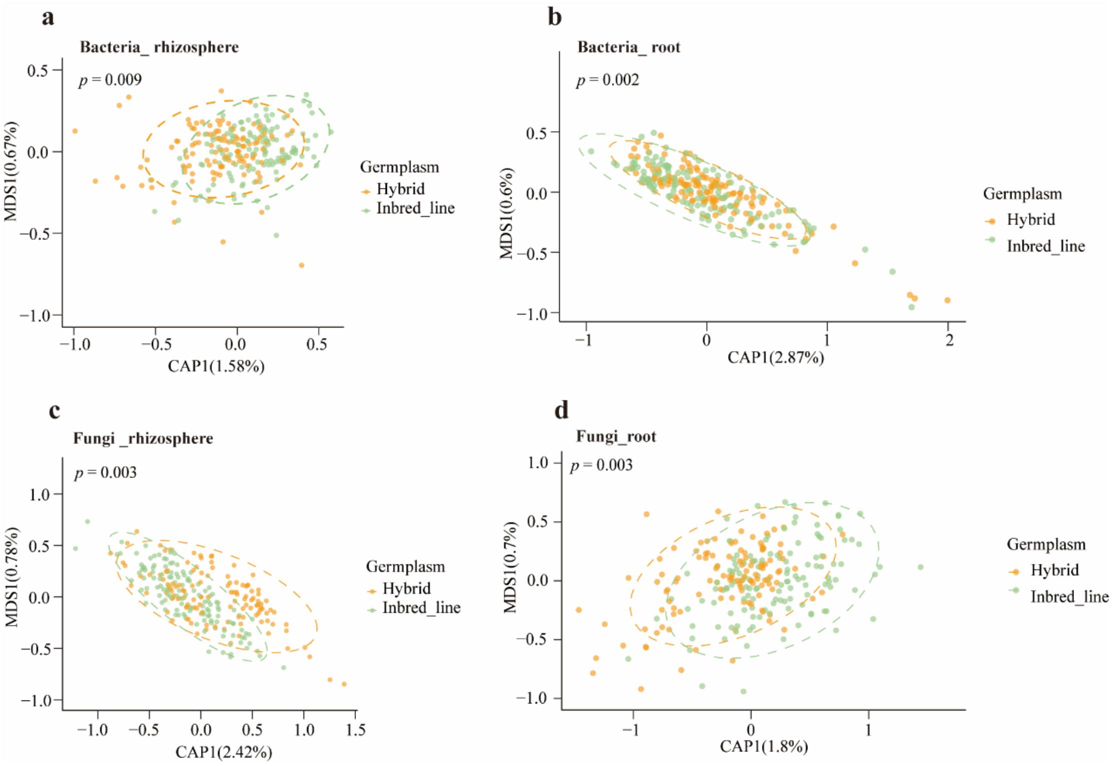

Supplementary Figure 1. Constrained distance-based redundancy analysis revealed the difference of the microbiome composition (**a**, bacteria in rhizosphere; **b**, bacteria in root; **c**, fungi in rhizosphere; **d**, fungi in root) between inbred lines and hybrids. Two axes of variation in community composition (ordination of Bray-Curtis dissimilarity) are plotted for bacterial and fungal communities in maize root and rhizosphere. The CAP1 axis was constrained to capture variation explained by inbred/hybrid category; MDS1 was the dominant unconstrained axis of variation. ANOVA-like permutation tests were performed to assess statistical significance of the constrained axis. This data show that significant difference in microbiome community composition is observed for inbred line and hybrids regardless of bacteria and fungi in root and rhizosphere.

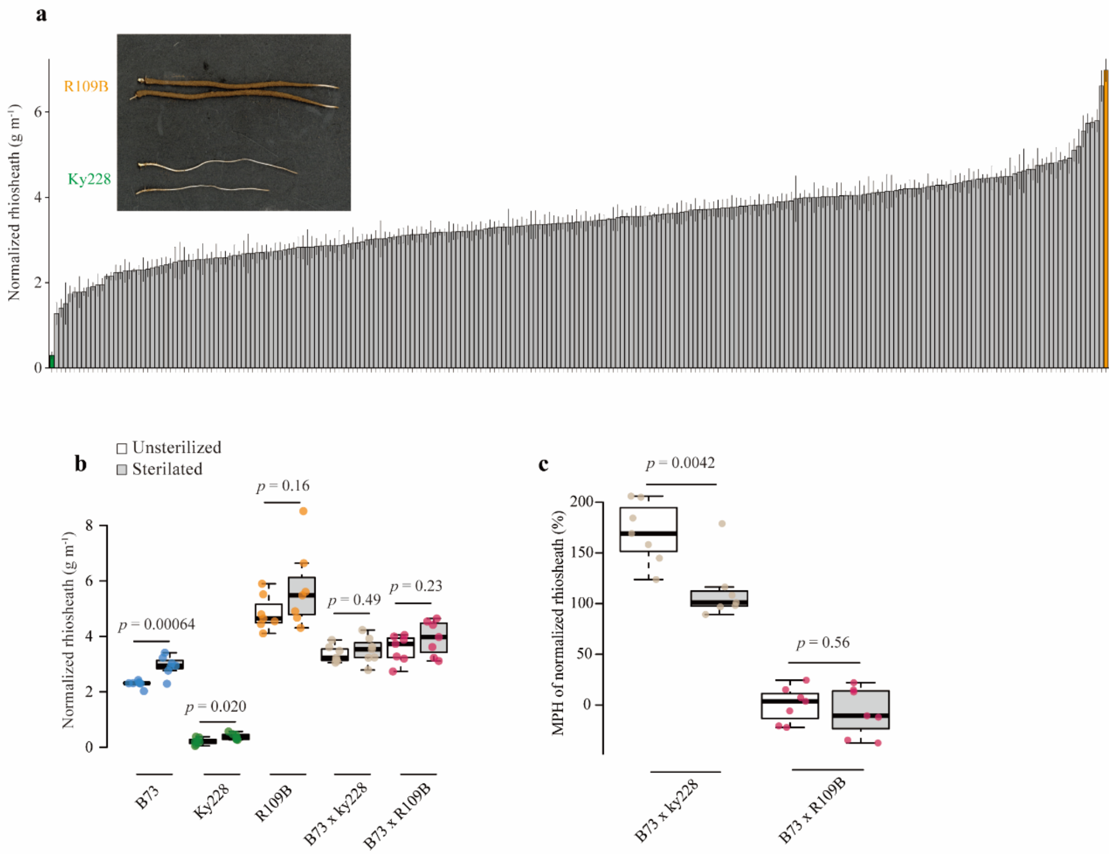

Supplementary Figure 2. Rhizosheath size showes heterosis in response to soil microbiome in primary root from 5-day maize seedlings.

a, Rhizosheath size performance among 233 maize genotypes collected from Maize 282 Association Panel; n ⩾ 3 biologically independent samples. Data are mean ± SEM. Ky228 (Green color) and R109B (Orange color) displays smallest and largest rhizosheath size, respectively. **b**, Rhizosheath performance of Ky228 and R109B in the presence or absence of soil microbiome. The data shows that R109B, B73 × Ky228, and B73 × R109B have larger rhizosheath size, while B73 and Ky228 have medium and smaller rhizosheath, respectively. Soil sterilization can significantly improve rhizosheath formation for the genotypes with smaller and medium rhizosheath size rather than that with larger rhizosheath, suggesting that the maize genotypes with larger rhizosheath are more adaptable to cope with environmental changes. **c**, The MPH (mid-parent heterosis) of rhizosheath size in the presence or absence of soil microbiome. **b** and **c**, n = 7 biologically independent samples. Significant differences among genotypes are indicated by exact *p* values (two-tailed Student's t-tests). Boxes span from the first to the third quartiles, centre lines represent the median values and whiskers show data lying within 1.5× the interquartile range of the lower and upper quartiles. Data points at the ends of whiskers represent outliers. This data shows that smaller-rhizosheath combination (B73 × Ky228) is more sensitive in response to soil microbiome. Rhizosheath size can be considered as a heritable key trait for crop breeding.

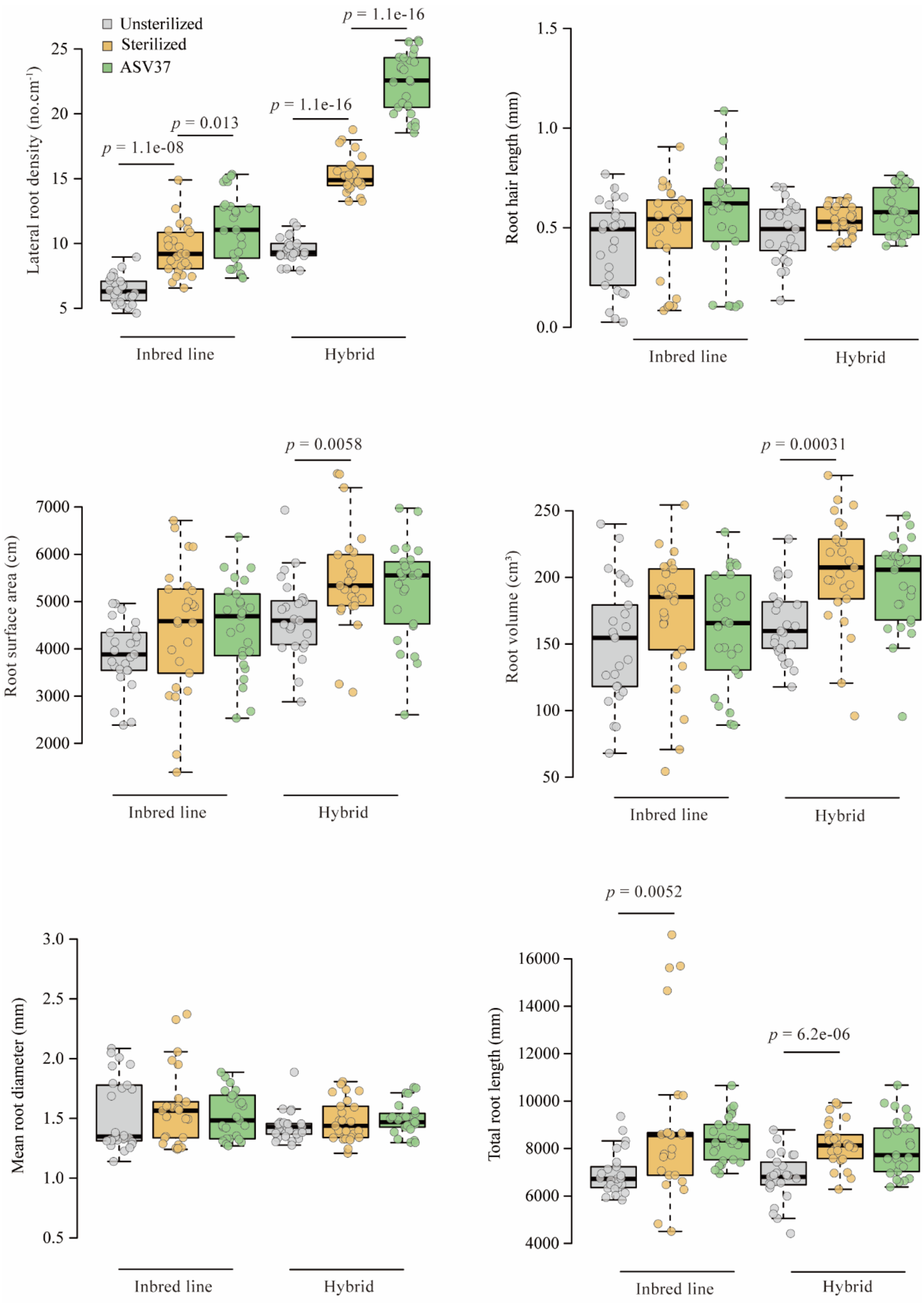

Supplementary Figure 3. Root traits performance in the presence or absence of soil microbiome and *Massilia* ASV37 inoculation treatments in nitrogen-poor soil. n = 25 biologically independent samples per treatment. Boxes span from the first to the third quartiles, centre lines represent the median values and whiskers show data lying within 1.5× the interquartile range of the lower and upper quartiles. Data points at the ends of whiskers

represent outliers. Significant differences at $p < 0.05$ among different treatments (Unsterilized vs Sterilized, Sterilized vs ASV37) are indicated by the exact p values (two-tailed Student's t-tests).

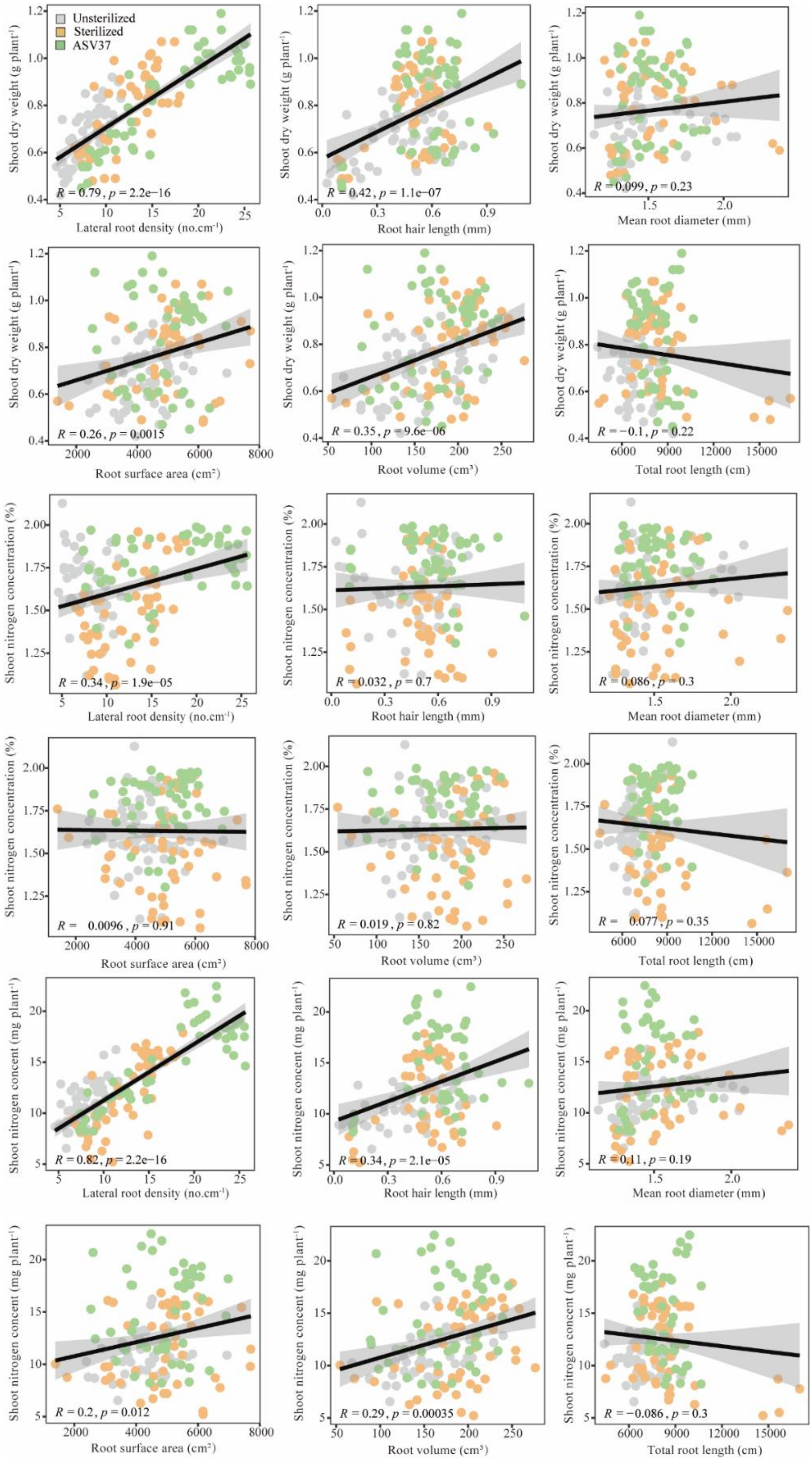

Supplementary Figure 4. Correlation between root traits and shoot performance in nitrogen-poor soil. Scatter plot with best-fit regression line (black solid) and 95% confidence interval (grey shading, two-sided Pearson correlation). The regression line represents the measure of centre, and the grey shading indicates the error band (95% confidence interval of the regression fit). n = 150 biologically independent samples.

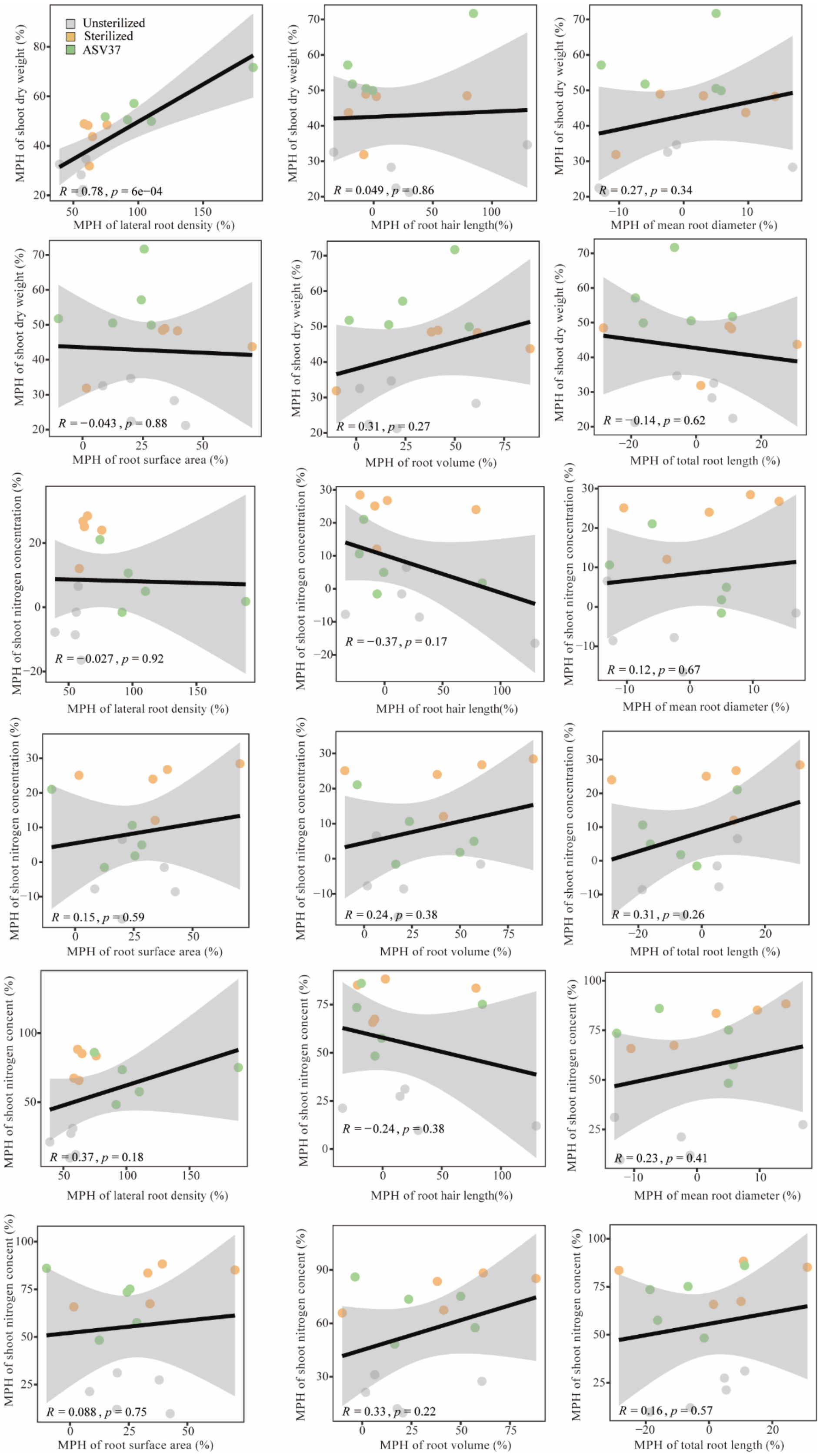
Unsterilized
Sterilized
ASV37
MPH of shoot dry weight (%)
R = 0.78 , p = 6e−04
MPH of lateral root density (%)
R = 0.049 , p = 0.86
MPH of root hair length(%)
R = 0.27 , p = 0.34
MPH of mean root diameter (%)
R = −0.043 , p = 0.88
MPH of root surface area (%)
R = 0.31 , p = 0.27
MPH of root volume (%)
R = −0.14 , p = 0.62
MPH of total root length (%)
MPH of shoot nitrogen concentration (%)
R = −0.027 , p = 0.92
R = −0.37 , p = 0.17
R = 0.12 , p = 0.67
R = 0.15 , p = 0.59
R = 0.24 , p = 0.38
R = 0.31 , p = 0.26
MPH of shoot nitrogen concent (%)
R = 0.37 , p = 0.18
R = −0.24 , p = 0.38
R = 0.23 , p = 0.41
R = 0.088 , p = 0.75
R = 0.33 , p = 0.22
R = 0.16 , p = 0.57

Supplementary Figure 5. Correlation between midparent heterosis (MPH) of root traits and MPH of shoot performance in nitrogen-poor soil. Scatter plot with best-fit regression line (black solid) and 95% confidence interval (grey shading, two-sided Pearson correlation). The regression line represents the measure of centre, and the grey shading indicates the error band (95% confidence interval of the regression fit), n = 25 biologically independent samples.

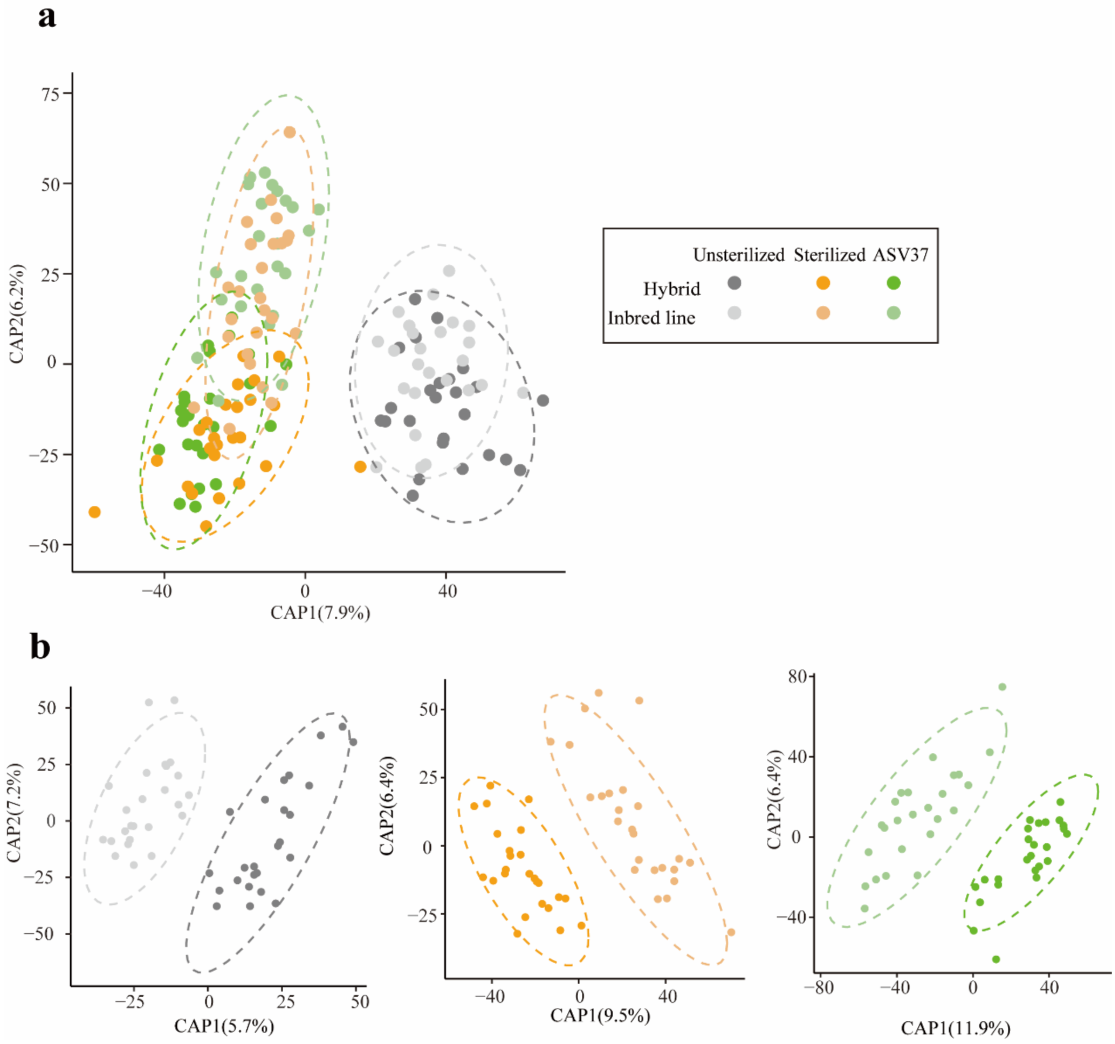

Supplementary Figure 6. Differential metabolite in the presence or absence of soil microbiome and *Massilia* inoculation treatments in poor-nitrogen soil. Differential metabolite screening by partial least squares discriminant analysis (PLS-DA) in all treatments (**a**) and each treatment (**b**). Score diagram of PLS-DA analysis model. Each point represents a sample; different colors represent different sample groups, and ellipses represent 95% confidence intervals. n = 25 biologically independent samples for each of the inbred line and hybrid groups per treatment.

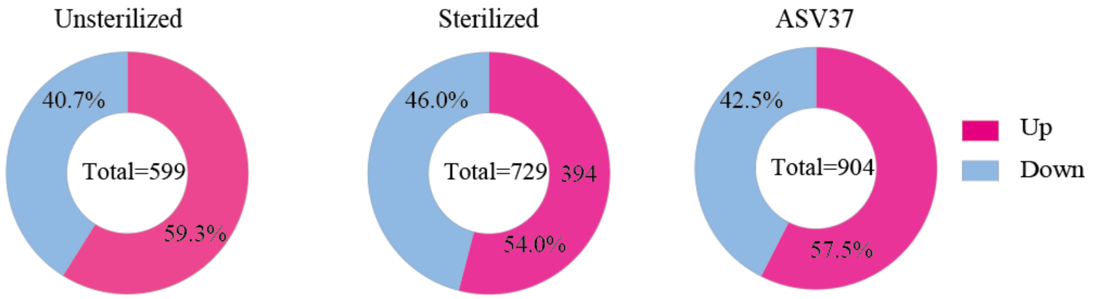

Supplementary Figure 7. Differential metabolites distributions in the presence or absence of soil microbiome and *Massilia* inoculation treatments in poor-nitrogen soil. Only the statistical significance metabolites pass screening criteria: (1) Variable Importance in the Projection (VIP) of Orthogonal Partial Least Squares Discriminant Analysis (OPLS-DA) model ≥ 1, (2) Fold Change ≥ 1.2 or ≤ 0.83, (3) p-value < 0.05, would be considered as differential metabolites.

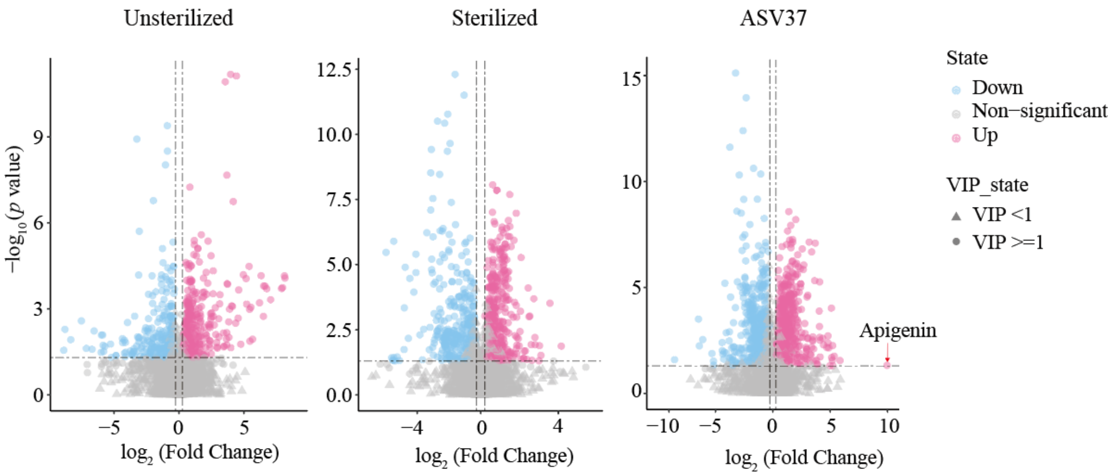

Supplementary Figure 8. Volcano plot showed the differential metabolites in the presence or absence of soil microbiome and *Massilia* inoculation treatments in poor-nitrogen soil.

Significance was determined using two-sided statistical tests, with adjusted *P* values shown as $-\log_{10}$(*p*-value). X-axis represents the $\log_2$ fold change and the y-axis represents the -$\log_{10}$ *p*-value of each metabolite. Blue dots indicate significantly down-regulated metabolites, red dots represent significantly up-regulated metabolites and gray dots are metabolites with no significant difference. Circle-shaped markers denote metabolites with a Variable Importance in Projection (VIP) score ≥ 1, while triangle-shaped markers represent metabolites with a VIP score < 1.

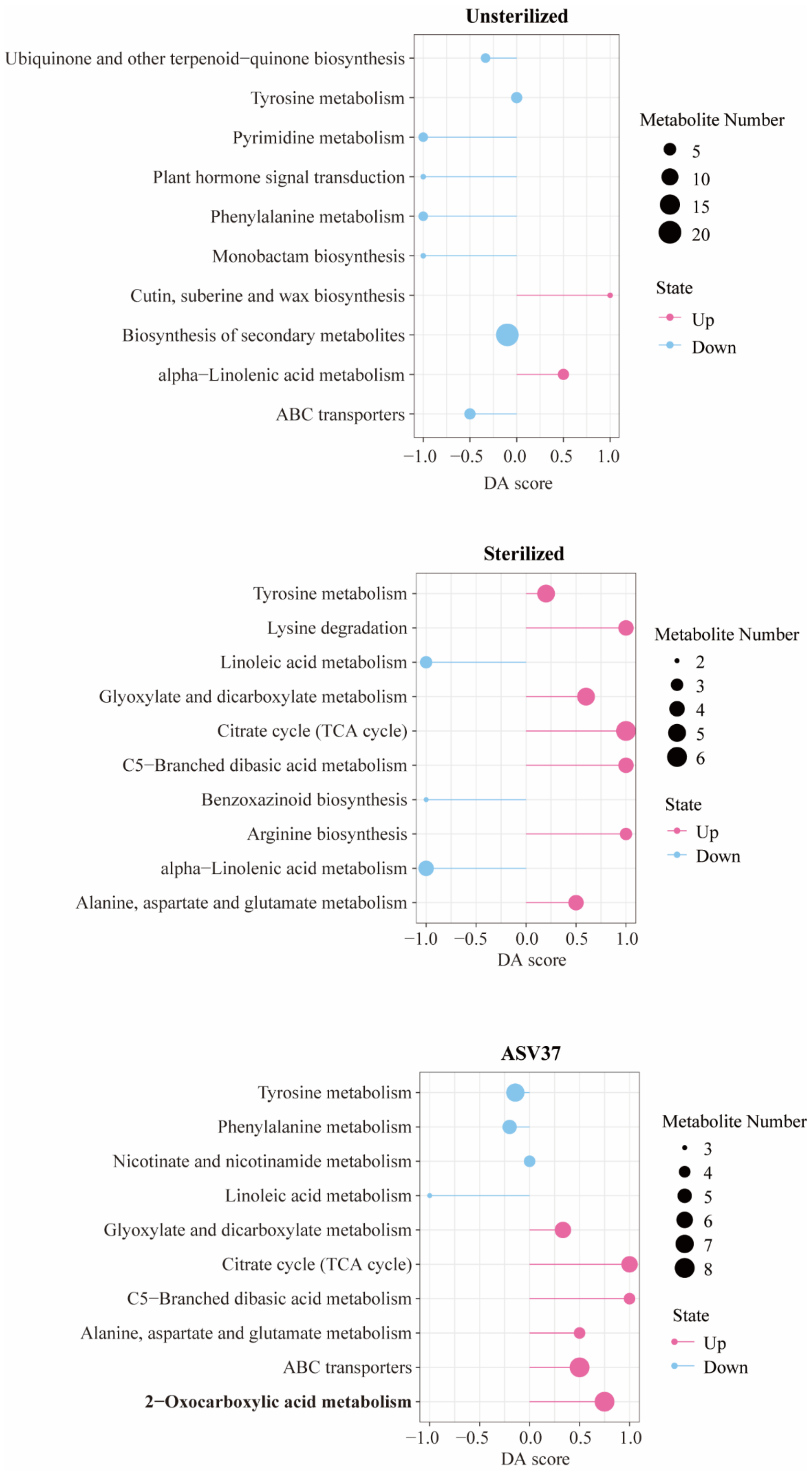

Unsterilized
Ubiquinone and other terpenoid−quinone biosynthesis
Tyrosine metabolism
Pyrimidine metabolism
Plant hormone signal transduction
Phenylalanine metabolism
Monobactam biosynthesis
Cutin, suberine and wax biosynthesis
Biosynthesis of secondary metabolites
alpha−Linolenic acid metabolism
ABC transporters
−1.0 −0.5 0.0 0.5 1.0
DA score
Metabolite Number
5
10
15
20
State
Up
Down
Sterilized
Tyrosine metabolism
Lysine degradation
Linoleic acid metabolism
Glyoxylate and dicarboxylate metabolism
Citrate cycle (TCA cycle)
C5−Branched dibasic acid metabolism
Benzoxazinoid biosynthesis
Arginine biosynthesis
alpha−Linolenic acid metabolism
Alanine, aspartate and glutamate metabolism
−1.0 −0.5 0.0 0.5 1.0
DA score
Metabolite Number
2
3
4
5
6
State
Up
Down
ASV37
Tyrosine metabolism
Phenylalanine metabolism
Nicotinate and nicotinamide metabolism
Linoleic acid metabolism
Glyoxylate and dicarboxylate metabolism
Citrate cycle (TCA cycle)
C5−Branched dibasic acid metabolism
Alanine, aspartate and glutamate metabolism
ABC transporters
2−Oxocarboxylic acid metabolism
−1.0 −0.5 0.0 0.5 1.0
DA score
Metabolite Number
3
4
5
6
7
8
State
Up
Down

Supplementary Figure 9. Metabolic pathway enrichment analysis based on the KEGG database revealed significantly altered metabolic pathways in the presence or absence of soil microbiome and *Massilia* inoculation treatments in poor-nitrogen soil. The Y-axis represents the name of the metabolic pathway, and the X-axis represents the differential abundance score (DA score). DA score is the overall total change of all metabolites in a metabolic pathway. DA score of 1 indicates that the expression trend of all annotated differential metabolites in the pathway is up-regulated, and a score of negative 1 indicates that the expression trend of all annotated differential metabolites in the pathway is downregulated. The length of the line segment represents the absolute value of the DA score, the size of the dot at the endpoint of the line segment represents the number of metabolites in the pathway, and the larger the dot, the greater the number of metabolites.

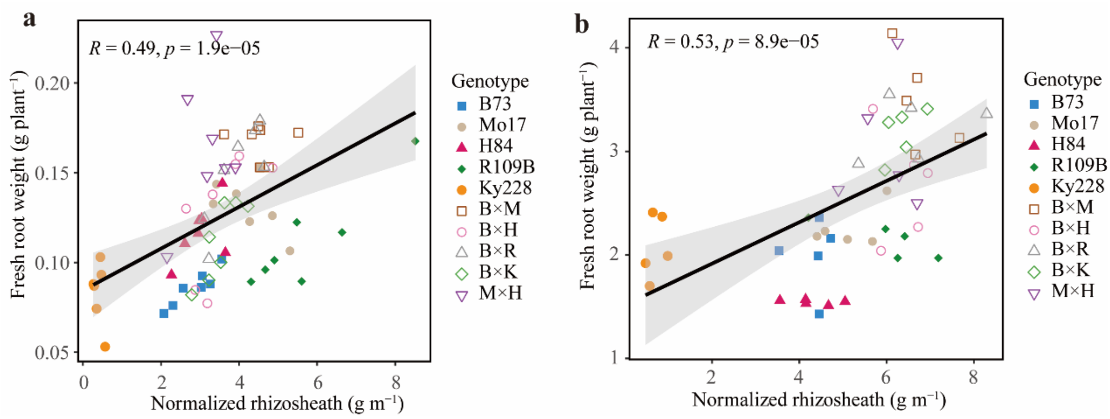

Supplementary Figure 10. The association between rhizosheath size and root weight for fresh primary root for 5-day seedings (a) and total fresh root weight for 4-week young seedings (b) in nitrogen-poor soil. n = 5 replicates per each genotype for total fresh root weight. Scatter plot with best-fit regression line (black solid) and 95% confidence interval (grey shading, two-sided Pearson correlation). The regression line represents the measure of centre, and the grey shading indicates the error band (95% confidence interval of the regression fit). Different solid and hollow shapes for data points represent inbred lines (B73, Mo17, H84, R109B, and Ky228) and hybrids (B×M, B×H, B×R, B×K, and M×H), respectively. This data suggests that rhizosheath size can respond well to the growth and development of maize root systems. B×M, B73×Mo17; B×H, B73×H84; B×R, B73×R109B; B×K, B73×Ky228; and M×H, Mo17×H84).

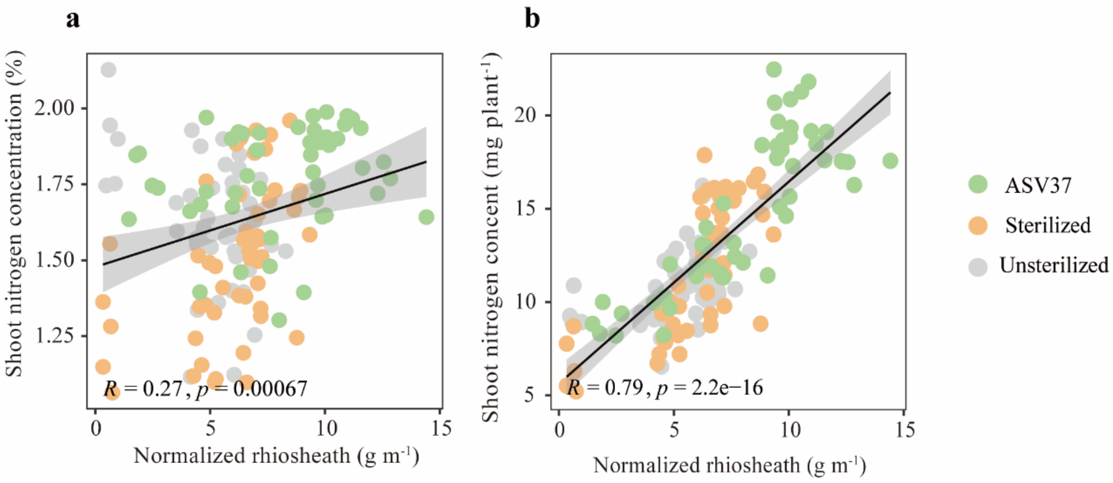

Supplementary Figure 11. Correlation between the rhizosheath size and shoot nitrogen concentration (**a**) and shoot nitrogen content (**b**) performance in nitrogen-poor soil. Scatter plot with best-fit regression line (black solid) and 95% confidence interval (grey shading, two-sided Pearson correlation). The regression line represents the measure of centre, and the grey shading indicates the error band (95% confidence interval of the regression fit). n = 150 biologically independent samples.

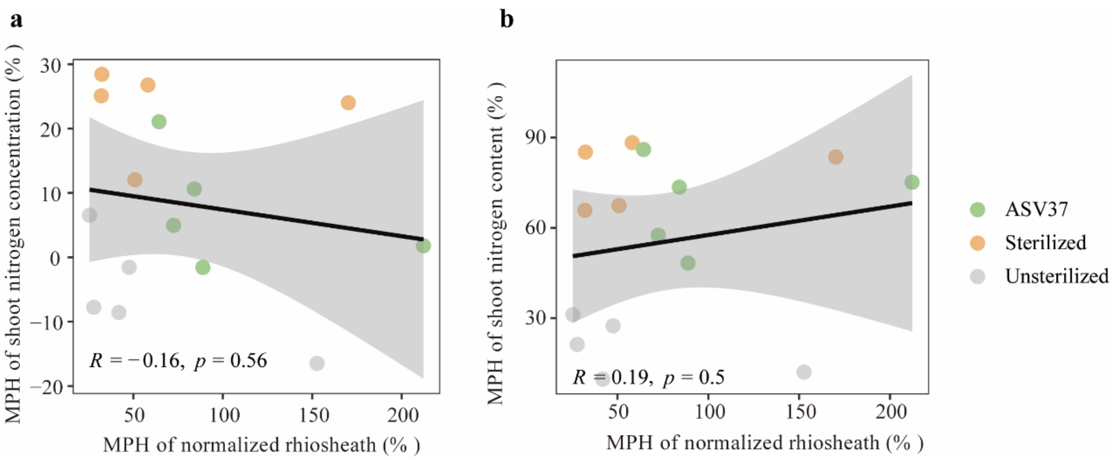

Supplementary Figure 12. Correlation between the midparent heterosis (MPH) of rhizosheath size and MPH of shoot nitrogen concentration (**a**) and shoot nitrogen content (**b**) performance in nitrogen-poor soil. Scatter plot with best-fit regression line (black solid) and 95% confidence interval (grey shading, two-sided Pearson correlation). The regression line represents the measure of centre, and the grey shading indicates the error band (95% confidence interval of the regression fit). n = 25 biologically independent samples.

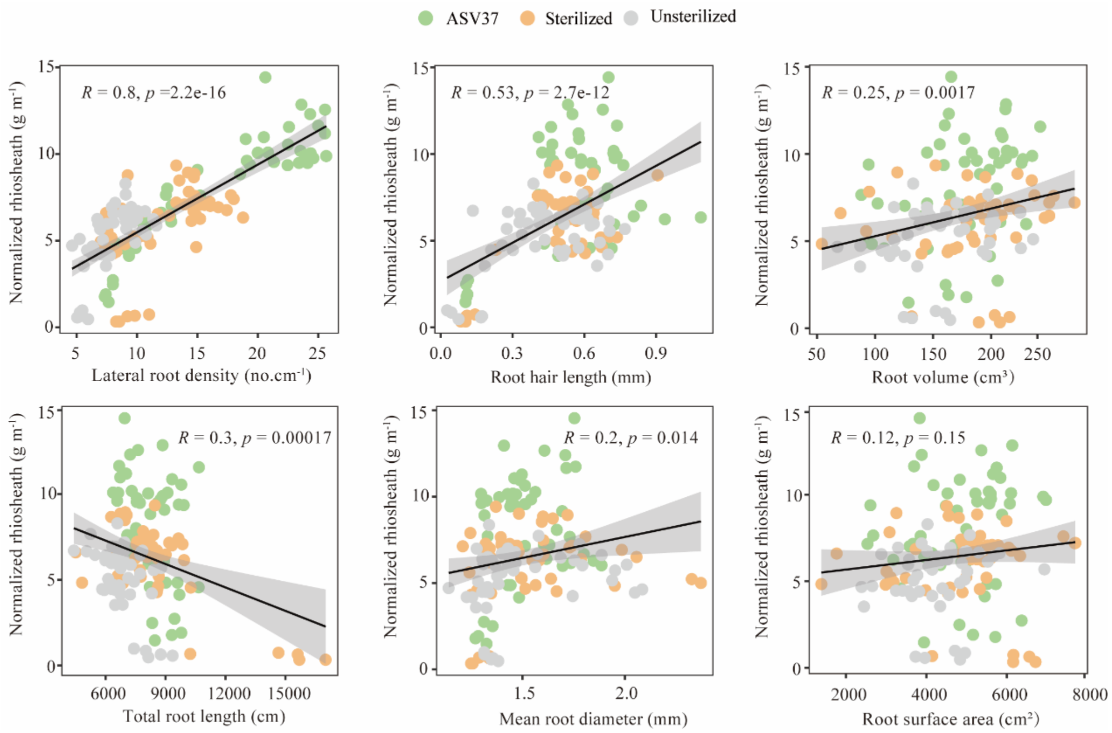

Supplementary Figure 13. Correlation between the rhizosheath size and root traits performance in nitrogen-poor soil. Scatter plot with best-fit regression line (black solid) and 95% confidence interval (grey shading, two-sided Pearson correlation). The regression line represents the measure of centre, and the grey shading indicates the error band (95% confidence interval of the regression fit). n = 150 biologically independent samples.

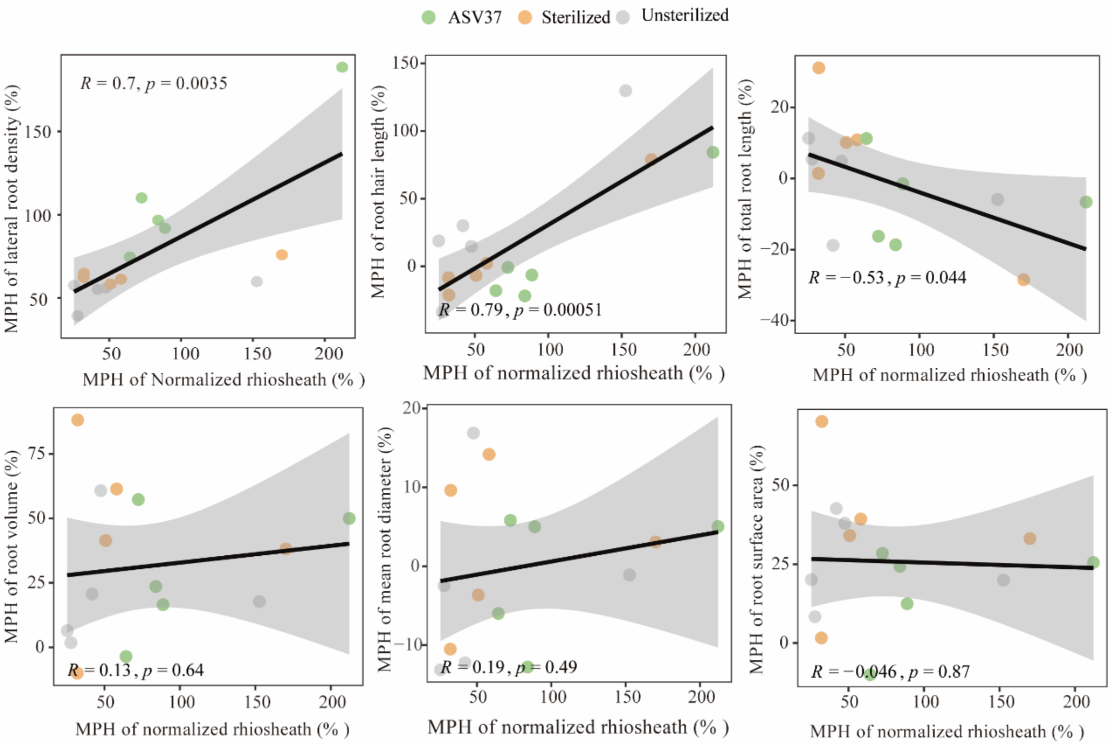

Supplementary Figure 14. Correlation between the midparent heterosis (MPH) of rhizosheath size and MPH of root traits performance in nitrogen-poor soil. Scatter plot with best-fit regression line (black solid) and 95% confidence interval (grey shading, two-sided Pearson correlation). The regression line represents the measure of centre, and the grey shading indicates the error band (95% confidence interval of the regression fit). n = 25 biologically independent samples.

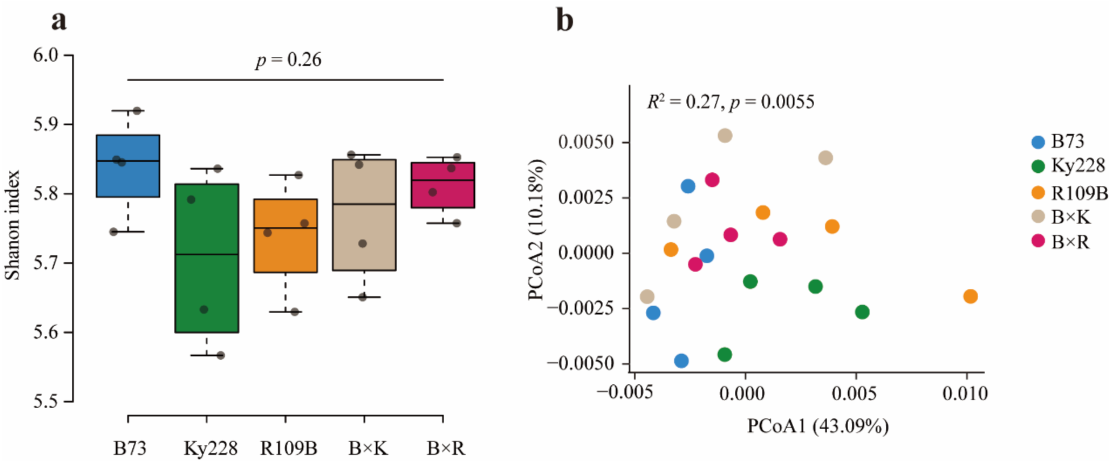

Supplementary Figure 15. Rhizosphere bacterial diversity for different genotypes in nitrogen-poor soil. a, Shannon index (α diversity) of rhizosphere samples of different genotypes in nitrogen-poor soil. n = 4 biologically independent sample. Significant differences at $p < 0.05$ among different genotypes are indicated by the exact p values (ANOVA, Tukey's HSD, two-sided). **b**, Distinct bacterial diversity associated with different genotypes rhizosphere microbiomes. Principal Coordinates Analysis (PCoA) using Bray–Curtis dissimilarity with PERMANOVA was applied to visualize significant differences in bacterial microbiota in the rhizosphere across five genotypes. Data points for bacteria (n = 20) are colour-coded according to the five genotypes. B×K, B73 × Ky228; B×R, B73 × R109B.

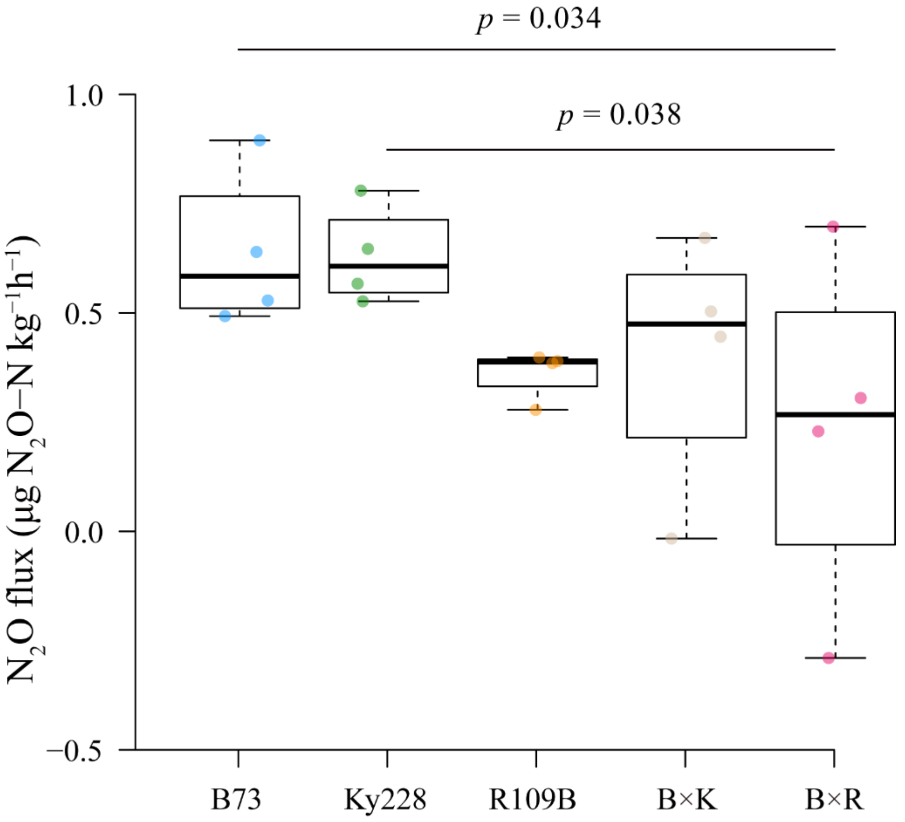

Supplementary Figure 16. N_2O emission (N_2O flux) of different genotypes in nitrogen-poor soil. n = 4 biologically independent sample. Significant differences at $p < 0.05$ among different genotypes are indicated by the exact p values (ANOVA, Tukey's HSD, two-sided). Boxes span from the first to the third quartiles, center lines represent median values and whiskers show data lying within 1.5× interquartile range of lower and upper quartiles. Data points at the ends of whiskers represent outliers. B×K, B73 × Ky228; B×R, B73 × R109B.

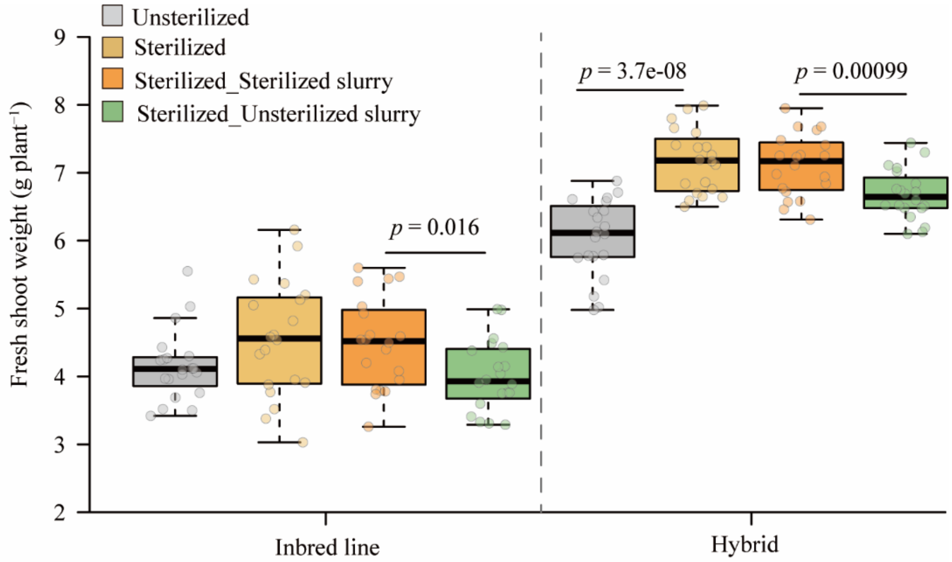

Supplementary Figure 17. Maize growth performance in the presence (Unsterilized) or absence (Sterilized) of soil microbiome and exogenous application of unsterilized slurry (Sterilized_ unsterilized slurry) or sterilized soil slurry (Sterilized_sterilized slurry) in nitrogen-poor soil. n = 25 biologically independent samples per treatment. Significant differences at $p < 0.05$ are indicated in response to in the presence or absence of soil microbiome and exogenous application of unsterilized slurry or unsterilized soil slurry by the exact *p* values (two-tailed Student's t-tests). Boxes span from the first to the third quartiles, center lines represent median values and whiskers show data lying within 1.5× interquartile range of lower and upper quartiles. Data points at the ends of whiskers represent outliers. This data suggests that soil sterilization improves plant growth by eliminating harmful microbes, not altering nutrient availability.

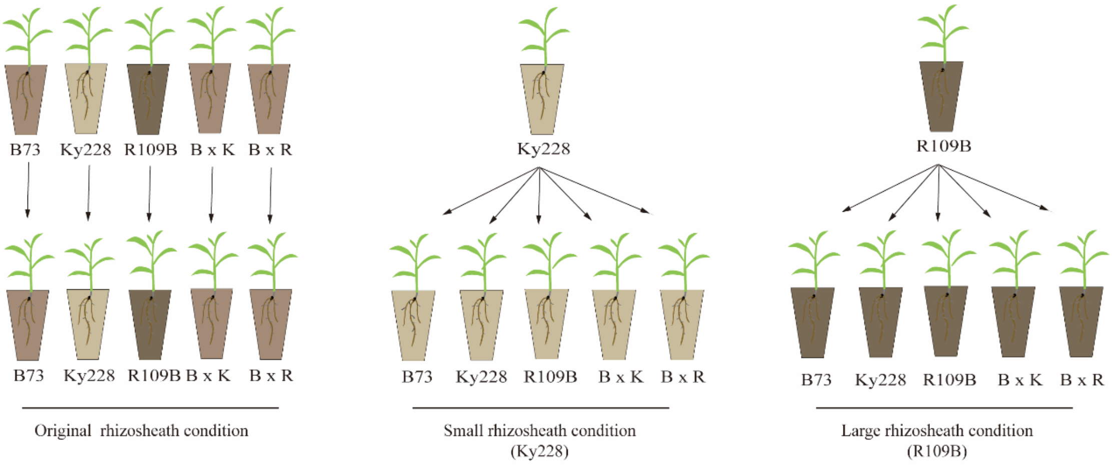

Supplementary Figure 18. Rhizosheath transplantation experiment. To investigate the influence of rhizosheath size on maize plant growth against low nitrogen stress, maize plants with different rhizosheath sizes were transplanted into nitrogen-deficient soil previously conditioned by either large-rhizosheath (genotype R109B) or small-rhizosheath (genotype Ky228) or original-rhizospeath maize plants to assess the impact of rhizosheath size on plant growth under nitrogen limitation. B×K, B73 × Ky228; B×R, B73 × R109B

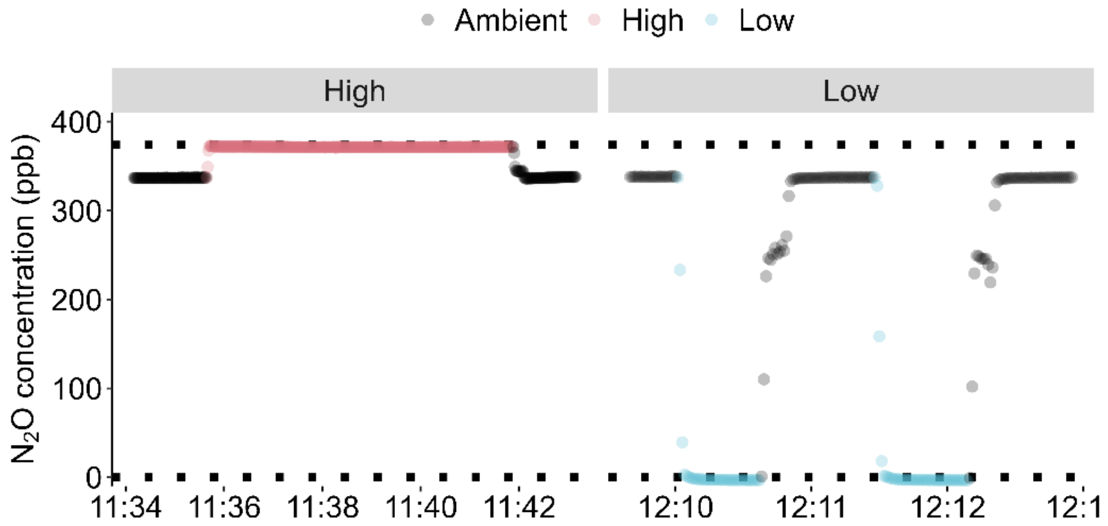

Supplementary Figure 19. Verification of the LI-7820 N_2O/H_2O analyzer using standard gases of 0 ppb (Low) and 374 ppb (High) N_2O. A two-point test covering the range of concentrations measured in our pot experiment confirmed the analyzer's accuracy. Our study focuses on relative differences in N_2O fluxes among genotypes rather than absolute fluxes, therefore, potential systematic errors do not affect the comparison between genotypes.
