# Supplementary material for: Rhizosheath inhabiting Massilia are linked to heterosis in roots of maize
Source: Nat Commun. 2025 Nov 28;16:10777. doi: 10.1038/s41467-025-65829-2 (PMC12663149; doi:10.1038/s41467-025-65829-2)
Supplement: Supplementary file 2 — Description of Additional Supplementary Files [file 41467_2025_65829_MOESM2_ESM.pdf]

Description of Additional Supplementary Files

File name: Supplementary Data 1

Description: Genetic materials selected in microbiome heterosis under different abiotic stress conditions.

File name: Supplementary Data 2

Description: Heterosis pattern of microbiome features (diversity index and ASVs) in both root and rhizosphere under different soil abiotic stresses.

File name: Supplementary Data 3

Description: Oxalobacteraceae isolates used for inoculation experiment.Three crossing triplets using different synthetic communities (SynCom) (e.g., Massilia ASV37 alone, a 17-member synthetic bacterial community (SynCom17) of Massilia isolates that did not include ASV37 and an 18-member Massilia (SynCom18) including SynCom17 and ASV37 conditions.

File name: Supplementary Data 4

Description: Shoot and root traits performce with and without soil microbiome and Massilia inoculation treatments in nitrogen-poor soil. n = 5 biologically independent samples per genotype.

File name: Supplementary Data 5

Description: The midparent heterosis (MPH) of shoot and root traits performce with and without soil microbiome and Massilia inoculation treatments in nitrogen-poor soil. n = 3 biologically independent samples per each conbination.

File name: Supplementary Data 6

Description: The correlation (Pearson correlation) among all shoot and root traits in nitrogen-poor soil.*0.01 < p ≤ 0.05; **0.001 < p ≤ 0.01; ***p ≤ 0.001.

File name: Supplementary Data 7

Description: Statistical significanceof the correlation (Pearson correlation) among the midparent heterosis (MPH) of all shoot and root traits in nitrogen-poor soil.*$0.01 < p \leqslant 0.05$; **$0.001 < p \leqslant 0.01$; ***$p \leqslant 0.001$.

File name: Supplementary Data 8

Description: 3,224 metabolic components were identified and annotated across all samples with and without soil microbiome and Massilia inoculation treatments in nitrogen-poor soil.

File name: Supplementary Data 9

Description: The count and percentage of metabolites in particular pathway over all metabolites with pathway categorial information.
