# Supplementary material for: Rhizosheath inhabiting Massilia are linked to heterosis in roots of maize
Source: Nat Commun. 2025 Nov 28;16:10777. doi: 10.1038/s41467-025-65829-2 (PMC12663149; doi:10.1038/s41467-025-65829-2)
Supplement: Supplementary file 4 — Reporting Summary [file 41467_2025_65829_MOESM4_ESM.pdf]

nature portfolio

Corresponding author(s): Xinping Chen, Frank Hochholdinger and Peng Yu

Last updated by author(s): Oct 3, 2025

Reporting Summary

Nature Portfolio wishes to improve the reproducibility of the work that we publish. This form provides structure for consistency and transparency in reporting. For further information on Nature Portfolio policies, see our Editorial Policies and the Editorial Policy Checklist.

Statistics

For all statistical analyses, confirm that the following items are present in the figure legend, table legend, main text, or Methods section.

n/a	Confirmed	
☐	☒	The exact sample size (*n*) for each experimental group/condition, given as a discrete number and unit of measurement
☐	☒	A statement on whether measurements were taken from distinct samples or whether the same sample was measured repeatedly
☐	☒	The statistical test(s) used AND whether they are one- or two-sided<br>*Only common tests should be described solely by name; describe more complex techniques in the Methods section.*
☐	☒	A description of all covariates tested
☐	☒	A description of any assumptions or corrections, such as tests of normality and adjustment for multiple comparisons
☐	☒	A full description of the statistical parameters including central tendency (e.g. means) or other basic estimates (e.g. regression coefficient) AND variation (e.g. standard deviation) or associated estimates of uncertainty (e.g. confidence intervals)
☐	☒	For null hypothesis testing, the test statistic (e.g. *F*, *t*, *r*) with confidence intervals, effect sizes, degrees of freedom and *P* value noted<br>*Give P values as exact values whenever suitable.*
☒	☐	For Bayesian analysis, information on the choice of priors and Markov chain Monte Carlo settings
☒	☐	For hierarchical and complex designs, identification of the appropriate level for tests and full reporting of outcomes
☐	☒	Estimates of effect sizes (e.g. Cohen's *d*, Pearson's *r*), indicating how they were calculated

Our web collection on statistics for biologists contains articles on many of the points above.

Software and code

Policy information about availability of computer code

Data collection	All these collected data was provided in the Supplemental Datasets.
Data analysis	The SSUrRNA database from SILVA database (release 138, 2020, https://www.arb-silva.de/) and UNITE database (v8.3, 2021, https://unite.ut.ee/) were used for analyzing the bacterial 16S and fungal ITS sequences, respectively. Pair-end reads were merged by FLASH (v1.2.7). Microbial sequence analysis by QIIME 2 software (v2020.6) and DADA2. Reads normalization by DESeq2 (v1.34.0).<br><br>High-performance liquid chromatography (HPLC) was used for analyzing the lyophilized metabolomic samples. Metabolites were separated and detected by Waters 2777c UPLC (Waters, USA) coupled with a Q Exactive HF high-resolution mass spectrometer (Thermo Fisher Scientific, USA). Mass spectrometry data were processed and identified using BGI's metabolome database, mzCloud, and the ChemSpider online database. Compound identification was performed through Compound Discoverer (v.3.3, Thermo Fisher Scientific, USA). The identified metabolites were classified, and functional annotation was carried out by comparing against the KEGG database to obtain KEGG IDs, categories, and associated pathways.<br><br>Metagenome assembly was performed using MEGAHIT. MetaGeneMark (http://exon.gatech.edu/meta_gmhmmp.cgi, Version 3.26) was used to identify coding regions in the genome. Redundancy in the gene data was reduced using MMseqs242 (https://github.com/soedinglab/mmseqs2, Version 12-113e3). eggNOG was used to classify genes into functional categories.<br><br>Boxplots generated by BoxPlotR. Statistical analyses by R (v4.1.0).

For manuscripts utilizing custom algorithms or software that are central to the research but not yet described in published literature, software must be made available to editors and reviewers. We strongly encourage code deposition in a community repository (e.g. GitHub). See the Nature Portfolio guidelines for submitting code & software for further information.

Data

Policy information about availability of data

All manuscripts must include a data availability statement. This statement should provide the following information, where applicable:

- Accession codes, unique identifiers, or web links for publicly available datasets
- A description of any restrictions on data availability
- For clinical datasets or third party data, please ensure that the statement adheres to our policy

Raw bacterial 16S and fungal ITS sequencing data for root and rhizosphere samples under different stress conditions have been deposited in the Sequence Read Archive (http://www.ncbi.nlm.nih.gov/sra) under BioProject ID PRJNA889703. Raw bacterial 16S sequencing data for rhizosheath size experiment and raw soil metagenomics sequencing data have been deposited under BioProject ID PRJNA1236007. Non-targeted metabolomics data has been deposited in the MetaboLights under BioProject ID MTBLS12613. All statistical data are provided with this paper. Source data are provided with this paper.

Research involving human participants, their data, or biological material

Policy information about studies with human participants or human data. See also policy information about sex, gender (identity/presentation), and sexual orientation and race, ethnicity and racism.

Reporting on sex and gender	*Use the terms sex (biological attribute) and gender (shaped by social and cultural circumstances) carefully in order to avoid confusing both terms. Indicate if findings apply to only one sex or gender; describe whether sex and gender were considered in study design; whether sex and/or gender was determined based on self-reporting or assigned and methods used.*<br>*Provide in the source data disaggregated sex and gender data, where this information has been collected, and if consent has been obtained for sharing of individual-level data; provide overall numbers in this Reporting Summary. Please state if this information has not been collected.*<br>*Report sex- and gender-based analyses where performed, justify reasons for lack of sex- and gender-based analysis.*
Reporting on race, ethnicity, or other socially relevant groupings	*Please specify the socially constructed or socially relevant categorization variable(s) used in your manuscript and explain why they were used. Please note that such variables should not be used as proxies for other socially constructed/relevant variables (for example, race or ethnicity should not be used as a proxy for socioeconomic status).*<br>*Provide clear definitions of the relevant terms used, how they were provided (by the participants/respondents, the researchers, or third parties), and the method(s) used to classify people into the different categories (e.g. self-report, census or administrative data, social media data, etc.)*<br>*Please provide details about how you controlled for confounding variables in your analyses.*
Population characteristics	*Describe the covariate-relevant population characteristics of the human research participants (e.g. age, genotypic information, past and current diagnosis and treatment categories). If you filled out the behavioural & social sciences study design questions and have nothing to add here, write "See above."*
Recruitment	*Describe how participants were recruited. Outline any potential self-selection bias or other biases that may be present and how these are likely to impact results.*
Ethics oversight	*Identify the organization(s) that approved the study protocol.*

Note that full information on the approval of the study protocol must also be provided in the manuscript.

Field-specific reporting

Please select the one below that is the best fit for your research. If you are not sure, read the appropriate sections before making your selection.

☒ Life sciences ☐ Behavioural & social sciences ☐ Ecological, evolutionary & environmental sciences

For a reference copy of the document with all sections, see nature.com/documents/nr-reporting-summary-flat.pdf

Life sciences study design

All studies must disclose on these points even when the disclosure is negative.

Sample size	No specific statistical methods were used to determine sample size for the experiments.<br>Instead, 11 inbred lines cover the main germplasm pools of maize breeding including the stiff-stalk, non-stiff stalk and tropical/subtropical.<br>2 maize genotypes with opposite rhizosheath sizes were selected from 233 maize genotypes selected from Maize 282 Association Panel with fully reflecting maize genetic diversity. All detailed information was provided with supplemental dataset and figures.
Data exclusions	We have performed the outlier detection analysis using specific model and have not found any outliers in our dataset.
Replication	The main pot experiment for microbiome sequencing was performed once with three independent biological replicates and each replicate included three randomly selected plant roots.<br>Rhizosheath size measurement, Metabolome and metagenomics sequencing were performed once with five independent biological replicates and each replicate included three randomly selected plant roots.

Randomization	Different maize genotypes were grown in the phytochamber with a split plot design and the pot distribution in each block was randomized.
Blinding	All root, rhizosphere and rhizosheath samples for all genotypes under different stress conditions were harvested blindly with only samples numbers without access to the genotype and treatment identities. Data analysis was blinded to genotypes and allowing them to choose each subject blindly. Data on biomass, nutrients analysis were acquired by personnel without genotype/treatment information.

Reporting for specific materials, systems and methods

We require information from authors about some types of materials, experimental systems and methods used in many studies. Here, indicate whether each material, system or method listed is relevant to your study. If you are not sure if a list item applies to your research, read the appropriate section before selecting a response.

Materials & experimental systems

n/a	Involved in the study
☒	☐ Antibodies
☒	☐ Eukaryotic cell lines
☒	☐ Palaeontology and archaeology
☒	☐ Animals and other organisms
☒	☐ Clinical data
☒	☐ Dual use research of concern
☐	☒ Plants

Methods

n/a	Involved in the study
☒	☐ ChIP-seq
☒	☐ Flow cytometry
☒	☐ MRI-based neuroimaging

Dual use research of concern

Policy information about dual use research of concern

Hazards

Could the accidental, deliberate or reckless misuse of agents or technologies generated in the work, or the application of information presented in the manuscript, pose a threat to:

No	Yes
☒	☐ Public health
☒	☐ National security
☒	☐ Crops and/or livestock
☒	☐ Ecosystems
☒	☐ Any other significant area

Experiments of concern

Does the work involve any of these experiments of concern:

No	Yes
☒	☐ Demonstrate how to render a vaccine ineffective
☒	☐ Confer resistance to therapeutically useful antibiotics or antiviral agents
☒	☐ Enhance the virulence of a pathogen or render a nonpathogen virulent
☒	☐ Increase transmissibility of a pathogen
☒	☐ Alter the host range of a pathogen
☒	☐ Enable evasion of diagnostic/detection modalities
☒	☐ Enable the weaponization of a biological agent or toxin
☒	☐ Any other potentially harmful combination of experiments and agents

Plants

Seed stocks	*Report on the source of all seed stocks or other plant material used. If applicable, state the seed stock centre and catalogue number. If plant specimens were collected from the field, describe the collection location, date and sampling procedures.*
Novel plant genotypes	*Describe the methods by which all novel plant genotypes were produced. This includes those generated by transgenic approaches, gene editing, chemical/radiation-based mutagenesis and hybridization. For transgenic lines, describe the transformation method, the number of independent lines analyzed and the generation upon which experiments were performed. For gene-edited lines, describe the editor used, the endogenous sequence targeted for editing, the targeting guide RNA sequence (if applicable) and how the editor was applied.*
Authentication	*Describe any authentication procedures for each seed stock used or novel genotype generated. Describe any experiments used to assess the effect of a mutation and, where applicable, how potential secondary effects (e.g. second site T-DNA insertions, mosiacism, off-target gene editing) were examined.*
